# Virtual reality-assisted rehabilitation for postoperative C5 palsy: a pilot exploratory randomized controlled trial

**DOI:** 10.1186/s12984-025-01716-7

**Published:** 2025-08-13

**Authors:** Kyung-Soo Suk, Jinyoung Park, Taeyeon Choi, Byung Ho Lee, Yongjin Ahn, Sunung Yun, Si Young Park, Hak Sun Kim, Seoung-Hwan Moon, Ji-Won Kwon

**Affiliations:** 1 Department of Orthopedic Surgery, Yonsei University College of Medicine, 50 Yonsei-ro, Seodaemun-gu, Seoul, 03722 Republic of Korea; 2 Department of Rehabilitation Medicine, Gangnam Severance Hospital, Yonsei University College of Medicine, Seoul, Korea

**Keywords:** Virtual reality rehabilitation, C5 palsy, Postoperative recovery, Electromyography, Cervical surgery

## Abstract

**Background:**

C5 palsy is a debilitating complication that may occur after posterior cervical decompression or fusion surgery, characterized by acute deltoid and biceps weakness. While most cases resolve spontaneously, prolonged dysfunction imposes significant physical, psychological, and socioeconomic burdens. Virtual reality (VR) has emerged as a promising adjunct in neurorehabilitation, offering immersive environments that promote engagement and motor learning. However, its application in postoperative C5 palsy rehabilitation remains underexplored.

**Methods:**

This single-center randomized controlled trial was conducted from January to December 2023 at a tertiary academic hospital. Adult patients (≥ 20 years) who developed new-onset C5 palsy after posterior cervical fusion were enrolled. C5 palsy was defined as a ≥ 2-grade drop in shoulder flexion or abduction strength postoperatively. Patients were randomly assigned to either a control group that received standard postoperative rehabilitation or a VR-assisted group that received the same standard rehabilitation plus an additional VR-based rehabilitation program. VR rehabilitation included interactive, game-based shoulder exercises delivered via head-mounted displays during initial hospitalization and follow-ups at 3, 6, 12, and 24 weeks. Primary outcomes were surface electromyography-derived maximal voluntary isometric contraction (MVIC), %MVIC, and fatigue index (FI) of the deltoid muscles. Secondary outcomes included the Medical Research Council (MRC) scale, Neck Disability Index (NDI), EuroQoL-5 Dimension (EQ-5D), Visual Analog Scale (VAS), and Hospital Anxiety and Depression Scale (HADS). Data were collected preoperatively and at each postoperative visit. Ten patients (VR = 4, Control = 6) completed the study.

**Results:**

Final analysis included data from 4 patients in the VR group and 6 patients in the control group. The VR group demonstrated significantly greater efficiency in muscle activation, evidenced by lower %MVIC values at 24 weeks during both shoulder flexion (median 1.0 vs. 1.5; *p* = 0.025) and abduction (0.9 vs. 1.8; *p* = 0.014). Improvements in patient-reported quality of life (EQ-5D, *p* = 0.032) and arm pain reduction (VAS, *p* = 0.048) were observed. Depression scores (HADS-D) and anxiety scores (HADS-A) also trended lower in the VR group, particularly at 24 weeks (HADS-D: 4.0 vs. 9.5; *p* = 0.067). Functional metrics, including maximum arm elevation (from 90.0 cm to 145.0 cm) and apple placement count (from 25 to 55 per session), improved markedly in the VR group.

**Conclusions:**

VR-assisted rehabilitation may contribute to improved neuromuscular efficiency, pain reduction, and psychological well-being in patients with postoperative C5 palsy. These preliminary findings suggest that immersive VR could be a promising adjunct in postoperative spinal rehabilitation, warranting further investigation in larger studies.

**Trial registration:**

Clinical Research Information Service (CRIS), KCT0010436. Registered on April 21, 2025. *Retrospectively registered.*

**Supplementary Information:**

The online version contains supplementary material available at 10.1186/s12984-025-01716-7.

## Introduction

### Background

C5 palsy is a common complication of cervical spine decompression surgery, characterized primarily by deltoid and biceps muscle weakness, often accompanied by sensory deficits or radicular pain [1]. The incidence of C5 palsy varies widely, with reports ranging from 1 to 30%, depending on the surgical approach and patient population [2, 3]. This condition typically manifests shortly after surgery and may take several months to resolve, causing significant anxiety and stress for patients, their families, and clinicians [4]​. Clinically, C5 palsy is significant owing to its impact on patients’ ability to perform daily activities, particularly those involving shoulder abduction and flexion. Although the prognosis for recovery is generally favorable, with most patients showing substantial improvement within 6 months, its pathogenesis remains unclear [4–6]. Proposed mechanisms include direct nerve root injury during surgery, traction injury from spinal cord shift, ischemia due to reduced blood supply, and reperfusion injury [7, 8]. The socioeconomic impact of C5 palsy is considerable [9]. Prolonged periods of disability necessitate ongoing medical care and rehabilitation, which incur substantial costs. Additionally, the psychological burden on patients and their families, combined with the increased demand for healthcare resources, highlights the need for effective rehabilitation strategies to expedite recovery and alleviate anxiety [5, 10]. Given these challenges, there is a critical need for rehabilitation programs that support not only physical recovery but also psychological well-being.

Recent technological advancements have introduced innovative solutions, with virtual reality (VR) emerging as a promising tool in the medical field [11–14]. VR is increasingly being used across various healthcare domains, including pain management, psychiatric therapy, and physical rehabilitation. The immersive and interactive nature of VR enhances patient engagement, providing a more stimulating and motivating environment for rehabilitation [14, 15]. Several studies have explored the use of VR in medical rehabilitation, reporting positive outcomes [16–18]. For example, VR has been effectively used to manage chronic pain by diverting patients’ attention away from discomfort, thereby reducing perceived pain levels [14]. Additionally, VR-based therapy has shown benefits in treating mental health conditions such as anxiety and post-traumatic stress disorder by creating controlled, immersive environments where patients can safely confront and manage their symptoms [15].

### Rationale

In physical rehabilitation, VR offers unique advantages. It can simulate various real-world scenarios and provide instant feedback, which is crucial for motor learning and recovery [19]. VR-based rehabilitation has the potential to shorten recovery times and improve functional outcomes by making exercises more engaging and less monotonous. This is particularly relevant for conditions such as C5 palsy, where sustained patient motivation is essential for effective rehabilitation. This study aimed to evaluate the efficacy of VR-assisted rehabilitation in patients with C5 palsy following cervical spine surgery. By comparing VR-assisted rehabilitation with conventional methods, we sought to determine whether VR provides superior outcomes in motor function recovery and psychological well-being. Specifically, this study was designed to contribute to the growing body of evidence supporting the integration of VR technology in medical rehabilitation, thereby offering a potential solution to enhance patient care and reduce the socioeconomic burden associated with postoperative complications such as C5 palsy.

In summary, this study investigated the application of VR technology in the rehabilitation of patients with C5 palsy, addressing the critical need for innovative, effective, and engaging rehabilitation programs. Through this research, we aimed to demonstrate the potential benefits of VR in accelerating recovery, reducing healthcare costs, and improving the overall quality of life for patients affected by this challenging condition.

## Patients/Methods

### Study design and setting

This study was designed as a prospective, randomized controlled trial conducted at a tertiary medical institution to assess the efficacy of VR-assisted rehabilitation in patients with C5 palsy following posterior cervical spine surgery. The study was approved by the institutional review board (approval number: 3-2022-0070), and all patients provided written informed consent.

### Participants

Participants included adults aged 20 years and older scheduled for posterior cervical spinal fusion due to degenerative cervical conditions. The inclusion criteria were confirmed cervical spinal stenosis, cervical myelopathy, or recurrent cervical disc herniation unresponsive to conservative treatment for at least 3 months. Exclusion criteria encompassed patients with spinal or intervertebral disc infections, spinal tumors, active chronic infections, or human immunodeficiency virus (HIV), bleeding disorders, pre-existing shoulder conditions limiting abduction, or those unable to comply with follow-up visits. Surgeries were performed by an orthopedic spine surgeon with over 25 years of post-fellowship experience at a single tertiary hospital between January 2023 and December 2023. Patients who developed C5 palsy postoperatively were enrolled in the study.

### Randomization

Randomization was conducted using a block randomization method to ensure equal allocation between the VR and control groups. A research nurse, who was not involved in patient enrollment, outcome assessment, or data analysis, generated the random allocation sequence in advance using the RAND() function in Microsoft Excel. The sequence was securely stored and accessed only after determining patient eligibility postoperatively, ensuring allocation concealment and minimizing selection bias.

### Electrodiagnosis

Electrodiagnostic testing was selectively conducted approximately three weeks postoperatively in patients with bilateral weakness, atypical clinical progression, or when other potential causes, such as motor neuron disease or neuromuscular disorders unrelated to cervical spine pathology, needed to be ruled out following clinical confirmation of motor deterioration. This approach is not routinely applied to all cases of C5 palsy, but was used to exclude complex or unrelated neuromuscular conditions that could confound the results or be unevenly assigned to one group. Electrodiagnosis was conducted by a neurophysiologist with over 10 years of experience in nerve conduction studies and needle electromyography (EMG). Nerve conduction studies primarily assessed distal conduction of the median and ulnar nerves, with additional nerves examined on the basis of clinical suspicion. Needle EMG was performed on both paraspinal muscles at the C5-T1 spinal levels, as well as the rhomboid major, infraspinatus, supraspinatus, triceps, biceps, brachioradialis, flexor carpi radialis, abductor pollicis brevis, first dorsal interossei, and other relevant muscles on the affected side [20].

### Surface electromyography

Surface EMG measurements, including MVIC, %MVIC, and FI, were collected only at preoperatively and at 24 weeks postoperatively to assess long-term muscle activation and fatigue recovery by an experienced physical therapist using a surface EMG device (BTS FREEEMG 1000; BTS Bioengineering, Lombardia, Italy). The device had a bandpass filter of 20–500 Hz, a notch filter of 60 Hz, and a sampling rate of 1,024 Hz.

Before the EMG assessment, the active range of motion for shoulder flexion and abduction was measured. The skin surface over the anterior and middle deltoid muscles was cleaned with alcohol, and hair was removed to minimize skin impedance. Bluetooth-enabled wireless surface EMG probes were attached parallel to the muscles, following established guidelines, including the Surface Electromyography for the Non-Invasive Assessment of Muscles (SENIAM) and the Anatomical Guide for the Electromyographer [21, 22]. The active electrode was positioned 5 cm distal to the acromion along the anterior margin of the anterior deltoid and at the greatest bulge of the middle deltoid, along the line between the acromion and the lateral epicondyle of the elbow. Surface EMG in this study was not intended as a diagnostic tool for C5 palsy but rather as a means to quantitatively evaluate neuromuscular recovery and task-oriented muscle activation during rehabilitation. Although surface EMG is not part of the standard diagnostic workflow for C5 palsy, it has been increasingly used in spinal cord injury and peripheral nerve disorders as a non-invasive biomarker of residual motor function and neural reorganization during recovery [23, 24].

1. Maximal voluntary isometric contraction (MVIC).

Patients were seated on a stationary stool and positioned with their shoulders externally rotated to 45°. They were instructed to abduct their shoulder to 50% of their active range of motion, exerting maximal effort against the therapist’s resistance for 5 s. Surface EMG signals were recorded bilaterally from the anterior and middle deltoid muscles using wireless electrodes placed according to SENIAM guidelines. MVIC was calculated as the root mean square of the middle 3 s of the EMG signal, excluding the first and last seconds. Data analysis was performed using BTS EMG-Analyzer software (BTS Bioengineering). Each measurement was repeated three times with a 30-second rest interval, and the average of these measurements was reported [25, 26].

2) Percent maximal voluntary isometric contraction (%MVIC).

After a 1-minute rest following MVIC assessments, patients were instructed to abduct their shoulder in the same position as aforementioned while holding a 1-kg weight and maintaining 50% of their active range of motion for 5 s to measure the task-oriented EMG activity. The root mean square of the middle 3 s of the EMG signal was analyzed, and %MVIC was calculated as the ratio of task-oriented EMG activity to MVIC. Each task was performed three times with a 30-second rest interval, and the average %MVIC was calculated [27, 28].

$$ \% MVIC{\text{ }}\left( \% \right){\mkern 1mu} = {\mkern 1mu} \frac{{Task - oriented{\text{ }}EMG{\text{ }}activity}}{{MVIC}} \times 100 $$

A lower %MVIC indicated greater efficiency in completing tasks with reduced muscle activation.

3) Fatigue index (FI).

Muscle endurance and contraction strength were evaluated using the FI. To evaluate muscle fatigue, FI was measured bilaterally from the anterior and middle deltoid muscles preoperatively and from the affected side at 24 weeks postoperatively. Patients performed the MVIC task for 1 min with resistive shoulder flexion (anterior deltoid) and abduction (middle deltoid). The median frequency (MDF) of the EMG signal for the initial 3 s (MDF_initial_) and final 3 s (MDF_final_) was calculated, excluding the first and last 3 s to minimize noise and variability. A reduction in MDF over the duration of sustained muscle contraction is a well-recognized indicator of localized muscle fatigue due to decreased muscle fiber conduction velocity and altered motor unit recruitment patterns [29, 30]. Thus, FI was calculated based on changes in MDF from the beginning to the end of contraction. FI was determined as follows [29, 31]:

$$ FI{\text{ }}\left( \% \right){\text{ }} = {\text{ }}\frac{{\left( {MDF_{{initial}} - MDF_{{final}} } \right)}}{{MDF_{{initial}} }} \times 100 $$

### Surgical procedure and definition of C5 palsy

All participants exhibited multilevel spinal cord compression with foraminal stenosis and a marked reduction in interpedicular foraminal height due to disc height loss. Surgical interventions included staged anterior and posterior cervical spine fusion or laminoplasty. For cases of suspected bony compression during the anterior approach, uncinate process resection was performed at the affected segments.

C5 palsy was identified as a postoperative reduction of two or more grades on the Medical Research Council (MRC) scale for shoulder flexion or abduction compared to the preoperative baseline. Strength assessments were performed daily during the inpatient period by trained orthopedic clinicians. The diagnosis of C5 palsy was made when a noticeable motor deficit developed during hospitalization, typically within the first 3 to 5 days after surgery.

### High-resolution VR equipment details

The immersive VR display system (VIVE Pro 1.0; HTC Corporation, Hong Kong, China) is a sophisticated VR headset featuring dual AMOLED 3.5-inch screens, each with a resolution of 1440 × 1600 pixels, delivering a combined total resolution of 2880 × 1600 pixels. The VR equipment included a headset, a controller (with an option to use either of the two provided), and two base stations. The controllers enhanced interactivity, enabling precise motion tracking and task execution in the virtual environment. The two base stations ensured accurate spatial tracking, covering a 5 × 5-m area. The headset operated at a refresh rate of 90 Hz with a 110-degree field of view, providing an immersive experience. It featured high-resolution certified audio capabilities, detachable headphones, and support for high-impedance headphones, ensuring high-quality sound. For sensory input tracking, the VR headset was equipped with a G-sensor (accelerometer), gyroscope, proximity sensor, and interpupillary distance sensor. It used SteamVR Tracking 1.0 (Valve Corporation, Bellevue, WAS, USA) for VR tracking software. The VR controllers were equipped with SteamVR Tracking 1.0 and included a multifunction trackpad, grip buttons, dual-stage triggers, system buttons, and menu buttons.

### VR gameplay rules, procedure, and follow-up

The experimental group underwent VR-assisted rehabilitation using a head-mounted display with a handheld motion-tracking controller and a sensor for round-relative height detection. The VR system featured an interactive game designed to improve shoulder abduction and flexion. Each VR exercise cycle lasted 5 min and involved a mission where participants placed virtual apples into baskets. The process was as follows. At the start, baskets appeared and moved across the screen. Participants were instructed to pick apples from a box and place them into the moving baskets. The baskets alternated their movement every 10 s, randomly switching between left-to-right and right-to-left directions. The successful placement of an apple in a basket caused the basket’s height to increase by 2 cm; otherwise, the height remained unchanged. During each 5-minute session, 60 baskets appeared: 30 at the upper level and 30 at the lower level. The upper baskets started 80 cm above the ground and could rise to a maximum of 160 cm based on success. Each successful apple placement increased the basket’s height by 2 cm, while failures kept the height constant. The lower baskets started 60 cm above the ground. With each successful apple placement, the basket moved up by 2 cm; unsuccessful attempts caused the basket to rise by 2 cm.

The VR program consisted of two modes: active VR rehabilitation, where patients used only the affected arm, and assisted VR rehabilitation, which allowed the use of the unaffected arm to assist the affected arm. Patients performed VR exercises daily for 1 h during their first postoperative week of hospitalization. Approximately 1 week after surgery, patients were discharged and subsequently returned for outpatient VR rehabilitation sessions at 3, 6, 12, and 24 weeks postoperatively. During these follow-up visits, the patients participated in 1-hour VR exercise sessions in a controlled environment. Data collected included the maximum height reached by the arm and the number of apples placed in the basket per cycle (Supplementary Video 1). Both the VR and control groups received standardized postoperative exercise education starting from the immediate postoperative period, once C5 palsy was confirmed. Education included daily passive and active shoulder range of motion (ROM) exercises, all performed in a standing position with feet shoulder-width apart. For passive ROM exercise, patients were instructed to use a self-assisted method by leaning their body and affected arm against a wall to facilitate elevation of the shoulder through the available range. The movement was performed in both flexion and abduction directions. Patients were guided to raise the arm as high as possible, hold the position for 10 s, lower the arm, and rest for 10 s. Each session consisted of 10 repetitions per movement direction, and five sessions were performed daily. For active ROM, patients actively elevated the affected shoulder in flexion and abduction to their maximum available range and held the position for up to 10 s, or as long as tolerable. To avoid sudden loss of muscle tension and reduce the risk of ligament or tendon strain, patients were instructed to support the arm with the contralateral (sound-side) hand when lowering it. Each session consisted of five repetitions with 30-second intervals, and five sessions were prescribed daily. This standardized protocol reflects real-world clinical practice, where patients typically perform unsupervised home-based rehabilitation guided by initial education.

### Primary outcomes and secondary outcomes

The primary outcome was the MVIC of the anterior and middle deltoid muscles, assessed using surface EMG data. Secondary outcomes included %MVIC, FI, MRC scales for affected shoulder flexion and abduction [32], and patient-reported outcomes, such as the Neck Disability Index (NDI) [33], EuroQol 5-Dimension Health Questionnaire (EQ-5D) [34], Visual Analog Scale (VAS) for neck and arm pain, and Hospital Anxiety and Depression Scale (HADS) [35].

The HADS consists of two subscales: HADS-A for anxiety and HADS-D for depression, each containing seven items scored from 0 to 3, with subscale scores summed to give a total ranging from 0 to 21. Higher scores indicate greater anxiety or depression, with scores above 10 suggesting clinically significant symptoms [36].

Data were collected at baseline and at 3, 6, 12, and 24 weeks postoperatively during outpatient follow-up visits. Secondary outcomes also incorporated functional assessments, such as the maximum height reached by the hand on the C5-palsy side and the number of apples placed in the basket during a single VR task cycle. These measures were compared between the “active” and “assisted” VR rehabilitation modes immediately postoperatively and at 3, 6, 12, and 24 weeks.

### Statistical analysis/study size

**Fig. 1:**
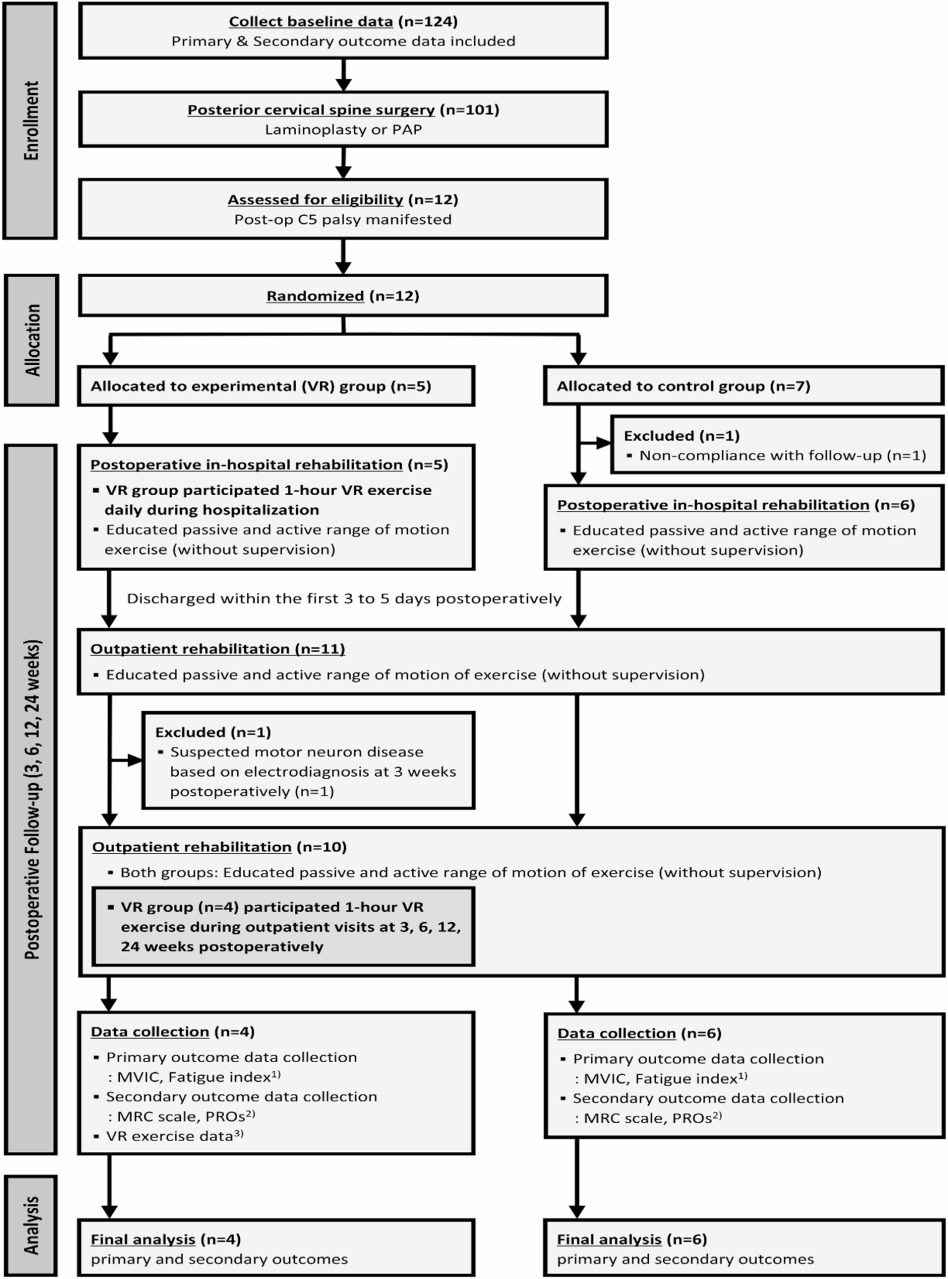
CONSORT flow diagram for the VR-assisted rehabilitation study. This diagram outlines the randomized controlled trial process, including participant enrollment (*N* = 12), randomization into two groups (Control Group: *N* = 7; VR Group: *N* = 5), exclusions due to ineligibility or loss to follow-up (*N* = 2), completion of follow-up, and data analysis. All randomized participants completed the follow-up period and were included in the final analysis. Assessments included: (1) MVIC and FI using dynamic EMG analysis and power spectrum analysis; (2) patient-reported outcomes (PROs) such as NDI, EQ-5D, VAS, HADS-A, HADS-D; and, (3) performance metrics, including maximum height achieved and the number of apples placed in the basket during VR tasks. *EQ-5D* EuroQol 5-Dimension health questionnaire, *FI* fatigue index, *HADS* Hospital Anxiety and Depression Scale, *PROs* patient-reported outcomes, *MVIC* maximum voluntary isometric contraction, *NDI* Neck Disability Index, *VAS* Visual Analogue Scale, *VR* virtual reality

This study was designed as an exploratory, preliminary investigation into the feasibility and potential effects of VR-assisted rehabilitation in patients with postoperative C5 palsy. Due to the rarity of C5 palsy following posterior cervical surgery, a formal a priori power calculation was not performed. Instead, a pragmatic sample size of five participants per group was targeted to assess feasibility and explore trends. Intergroup comparisons for continuous variables were performed using the Mann–Whitney U test, and categorical variables were compared using the chi-square test or Fisher’s exact test, as appropriate. The Wilcoxon signed-rank test was used to assess within-group differences over time. The primary outcome, maximal voluntary isometric contraction (MVIC), was assessed by calculating the 24-week/preoperative ratio. Given the small sample size, p-values were interpreted with caution. To further support the interpretation of between-group differences, Cohen’s d was calculated for key outcomes to estimate the magnitude of observed effects. Effect sizes were interpreted as small (d = 0.2), medium (d = 0.5), or large (d ≥ 0.8) according to standard conventions. This dual approach allows for a more nuanced interpretation of the data beyond statistical significance alone. Consequently, this study aimed to explore the potential effects of VR-assisted rehabilitation in a preliminary context, rather than to formally test a predefined hypothesis, due to the feasibility-oriented nature of the design and the limited patient population (Fig. 1).

## Results

### Incidence and demographics of C5 palsy

During the study period, 124 patients underwent preoperative screening and 101 underwent posterior cervical spine surgery, among whom patients with postoperative C5 palsy were considered for enrollment. A total of 12 patients were enrolled, with two excluded from the final analysis: one from the VR group and one from the control group. Although exclusions are typically performed prior to randomization, in this study, the exclusion of the VR group participant occurred three weeks after allocation, as abnormal findings suggestive of motor neuron disease were only detectable at that time through needle electromyography, which generally requires at least three weeks following the onset of neurological deficits. In the control group, one patient was excluded after allocation due to non-compliance with follow-up, which occurred before any intervention was initiated. Data from 10 patients (Control Group: *N* = 6; VR Group: *N* = 4) were analyzed. Baseline characteristics were comparable between the Control and VR groups, including age (68.5 years [48.0–78.0] and 53.5 years [42.0–61.0], respectively, *p* = 0.054), height, weight, and comorbidities, such as diabetes mellitus (66.7% and 0%, respectively, *p* = 0.076). The median number of operated segments was 4.0 for both groups (Control: 3.0–6.0 and VR: 4.0, *p* = 0.694). Posterior-anteroposterior (PAP) fusion surgery was performed in all participants, while resection of the uncinate process was conducted in 60% of cases (66.7% in the Control group and 50% in the VR group). The duration of surgery (239.0 min [207.0–292.0] and 280.0 min [221.0–327.0], respectively, *p* = 0.336) and estimated blood loss (450.0 mL [150.0–700.0] and 425.0 mL [200.0–550.0], respectively, *p* = 0.747) were similar between the Control and VR groups (Table 1). To complement these group-level summaries, individual baseline data for each participant are listed in Table 2.

**Table 1:** Demographic, surgical and postoperative data

Variable	Total (*N* = 10)	Control (*N* = 6)	VR (*N* = 4)	*P*
	*N* (%) or Median (range)			
Demographic data				
Sex (male, %)	10 (100.0)	6 (100.0)	4 (100.0)	> 0.999
Age (years)	61.0 (42.0–78.0)	68.5 (48.0–78.0)	53.5 (42.0–61.0)	0.054
Height (cm)	169.5 (160.0–190.0)	169.0 (163.0–171.7)	170.5 (160.0–190.0)	0.668
Weight (kg)	70.0 (65.0–100.0)	68.0 (65.0–91.9)	80.0 (68.0–100.0)	0.166
Diabetes mellitus	4 (40.0)	4 (66.7)	0 (0.0)	0.076
Hypertension	3 (30.0)	2 (33.3)	1 (25.0)	> 0.999
CVA	1 (10.0)	1 (16.7)	0 (0.0)	> 0.999
Surgical data				
Number of operated segments	4.0 (3.0–6.0)	4.0 (3.0–6.0)	4.0 (4.0–4.0)	0.694
Surgical procedure				
Resection of uncinate process	6 (60.0)	4 (66.7)	2 (50.0)	0.870
Surgical approach				
PAP	10 (100.0)	6 (100.0)	4 (100.0)	> 0.999
Surgical level				0.619
C3-6	1 (10.0)	1 (16.7)	0 (0.0)	
C3-7	6 (60.0)	2 (33.3)	4 (100.0)	
C3-T1	1 (10.0)	1 (16.7)	0 (0.0)	
C3-T2	1 (10.0)	1 (16.7)	0 (0.0)	
C4-T1	1 (10.0)	1 (16.7)	0 (0.0)	
Duration of surgery (minutes)	252.5 (207.0–327.0)	239.0 (207.0–292.0)	280.0 (221.0–327.0)	0.336
Estimated blood loss (mL)	450.0 (150.0–700.0)	450.0 (150.0–700.0)	425.0 (200.0–550.0)	0.747
Postoperative data				
Palsy side (right side, %)	4 (40.0)	2 (33.3)	2 (50.0)	> 0.999
MRC grade at the time of paralysis				
Shoulder flexion	2.0 (1.0–4.0)	2.0 (1.0–4.0)	1.75 (1.0–2.0)	0.640
Shoulder abduction	2.0 (1.0–2.0)	1.67 (1.0–2.0)	2.0 (2.0–2.0)	0.175

*CVA* cerebrovascular accident, *PAP* posterior-anteroposterior procedure, *MRC* Medical Research Council, *min* minimum, *max* maximum.

**Table 2:** Individual baseline data of the participants

Patient No.	Group	Sex	Age (yr)	Height (cm)	Weight (kg)	Past history			Surgical data						Postoperative data		
						DM	HTN	CVA	No. of operated segment	Surgical level	Approach	Resection of uncinate process	Duration of surgery (min)	Estimated blood loss (mL)	Palsy side	MRC grade at the time of paralysis	
																Shoulder flexion	Shoulder abduction
1	Control	M	48	172	92	+	–	–	4	C3-7	PAP	+	239	400	Left	1	2
2	Control	M	61	171	71	+	–	–	4	C3-7	PAP	+	292	700	Right	1	2
3	Control	M	67	165	67	+	–	–	5	C3-T1	PAP	-	237	700	Left	2	1
4	Control	M	70	169	65	+	+	+	4	C4-T1	PAP	+	280	500	Left	4	1
5	Control	M	77	163	66	–	+	–	6	C3-T2	PAP	-	207	400	Left	2	2
6	Control	M	78	169	69	–	–	–	3	C3-6	PAP	+	239	150	Right	2	2
7	VR	M	42	190	100	–	–	–	4	C3-7	PAP	+	266	300	Left	2	2
8	VR	M	47	160	68	–	+	–	4	C3-7	PAP	+	294	550	Right	2	2
9	VR	M	60	170	78	–	–	–	4	C3-7	PAP	–	327	550	Left	1	2
10	VR	M	61	171	82	–	–	–	4	C3-7	PAP	–	221	200	Right	2	2

For the reader’s convenience, patient numbers were ordered by group and age, rather than by enrollment sequence. All participants had a preoperative MRC grade of 5 for both shoulder flexion and abduction, indicating no muscle weakness in these movements prior to surgery.

*CVA* cerebrovascular accident, *DM* diabetes mellitus, *HTN* hypertension, *min* minute, *MRC* Medical Research Council, *VR* virtual reality group, *yr* year.

### Preoperative surface electromyography data

Prior to the initiation of any rehabilitation intervention, surface EMG data were collected from all participants to establish baseline muscle function. Preoperative MVIC values of the anterior deltoid during shoulder flexion were 415.2 µV (157.3–991.3) in the control group and 263.9 µV (92.1–545.8) in the VR group. The corresponding %MVIC values were 56.0% (14.2–81.4) and 74.4% (30.5–123.7), respectively. Similarly, for the middle deltoid, preoperative MVIC values were 422.5 µV (115.1–669.4) in the control group and 256.1 µV (154.8–628.1) in the VR group, with %MVIC values of 39.6% (11.6–61.1) and 47.3% (25.9–94.6). The FI of the anterior deltoid at baseline was 4.9% (1.9–5.5) in the control group and 3.9% (0.8–5.2) in the VR group. For the middle deltoid, the FI was 5.5% (–1.5–7.3) and 9.7% (1.1–10.2) for control and VR groups, respectively (Table 3).

**Table 3:** The effect of VR on muscle activities

		Preop					POD 24 wks					POD 24 wks/Preop			
Action	Variable	Control (*N* = 6)	VR (*N* = 4)	*P*	Cohen’s d		Control (*N* = 6)	VR (*N* = 4)	*P*	Cohen’s d		Control (*N* = 6)	VR (*N* = 4)	*P*	Cohen’s d
Shoulder flexion	MVIC_ant.del_	415.2 (157.3–991.3)	263.9 (92.1–545.8)	0.456	0.673		419.3 (151.5–663.9)	232.6 (48.1–425.3)	0.337	0.877		1.2 (0.2–2.9)	0.8 (0.5–1.0)	0.594	0.71
	%MVIC_ant.del_	56.0 (14.2–81.4)	74.4 (30.5–123.7)	0.241	0.701		86.8 (14.2–130.1)	75.4 (63.3–98.7)	0.594	0.152		1.5 (1.0–2.0)	1.0 (0.8–2.2)	0.337	0.456
	MVIC_mid.del_	422.5 (115.1–669.4)	256.1 (154.8–628.1)	0.915	0.233		408.3 (83.2–602.2)	409.9 (46.4–642.6)	0.915	0.004		1.0 (0.2–2.8)	0.8 (0.3–3.7)	0.915	0.001
	%MVIC_mid.del_	39.6 (11.6–61.1)	47.3 (25.9–94.6)	0.594	0.596		60.9 (11.7–79.1)	47.7 (17.5–67.3)	0.594	0.399		1.2 (1.0–2.0)	0.8 (0.7–1.1)	0.025*	1.64
	FI_ant.del_	4.9 (1.9–5.5)	3.9 (0.8–5.2)	0.551	0.526		-0.3 (-8.9–10.7)	3.4 (0.6–13.8)	0.594	0.651		-1.2 (-2.5–2.2)	3.6 (0.1–3.6)	0.136	1.269
Shoulder abduction	MVIC_ant.del_	417.8 (163.5–988.2)	266.4 (92.4–543.0)	0.456	0.679		420.3 (154.2–654.9)	234.2 (46.6–427.3)	0.337	0.874		1.2 (0.2–2.9)	0.8 (0.5–1.0)	0.594	0.7
	%MVIC_ant.del_	51.9 (43.9–129.8)	68.7 (36.2–105.9)	> 0.999	0.177		73.9 (59.8–136.6)	81.4 (52.5–95.1)	0.915	0.222		1.4 (0.7–2.6)	1.1 (0.9–1.9)	0.749	0.408
	MVIC_mid.del_	422.8 (115.0–737.1)	256.8 (155.0–633.9)	0.915	0.265		405.9 (86.8–603.4)	409.0 (46.4–655.1)	0.915	0.006		1.0 (0.2–2.7)	0.8 (0.3–3.6)	0.915	0.003
	%MVIC_mid.del_	45.0 (37.3–63.2)	68.3 (39.5–84.0)	0.11	1.145		91.9 (44.8–125.0)	60.0 (25.2–75.6)	0.11	1.268		1.8 (1.0–3.2)	0.9 (0.6–0.9)	0.014*	1.966
	FI_mid.del_	5.5 (-1.5–7.3)	9.7 (1.1–10.2)	0.371	0.499		6.0 (-14.8–18.0)	7.8 (2.2–10.5)	> 0.999	0.358		0.0 (-8.3–3.3)	1.0 (1.0–2.0)	0.766	0.625

Values are mean rank (min-max)

*MVIC* Maximum Voluntary Isometric Contraction (kg), *%MVIC* task-related EMG activity normalized to MVIC (%), *ant.del* anterior deltoid, *mid. del* middle deltoid, FI fatigue index, *POD* postoperative day, *Preop*. preoperative day 1, *wks* weeks. *VR* virtual reality

*, *P* < 0.05.

### Muscle strength and activities

Recovery of muscle strength, assessed via the MRC scale, showed substantial improvements in deltoid function in both groups by 24 weeks postoperatively. Median MRC grades for shoulder flexion and abduction reached 4.5–5.0, indicating near-complete recovery. No significant differences were observed in MRC grade recovery trajectories between the VR and Control groups. Surface EMG–based assessments revealed no significant group differences in absolute MVIC values during either shoulder flexion or abduction at baseline or 24 weeks. However, at 24 weeks, the %MVIC of the middle deltoid during flexion was significantly lower in the VR group than in the Control group (1.2 [1.0–2.0] vs. 0.8 [0.7–1.1], *p* = 0.025), indicating greater muscle efficiency. Similarly, the %MVIC ratio for shoulder abduction (POD 24 weeks/Preop) was significantly lower in the VR group than in the Control group (1.8 [1.0–3.2] vs. 0.9 [0.6–0.9], *p* = 0.014). FI, reflecting muscular endurance, remained comparable between groups at all time points (Table 3).

### Patient-reported outcomes

**Fig. 2:**
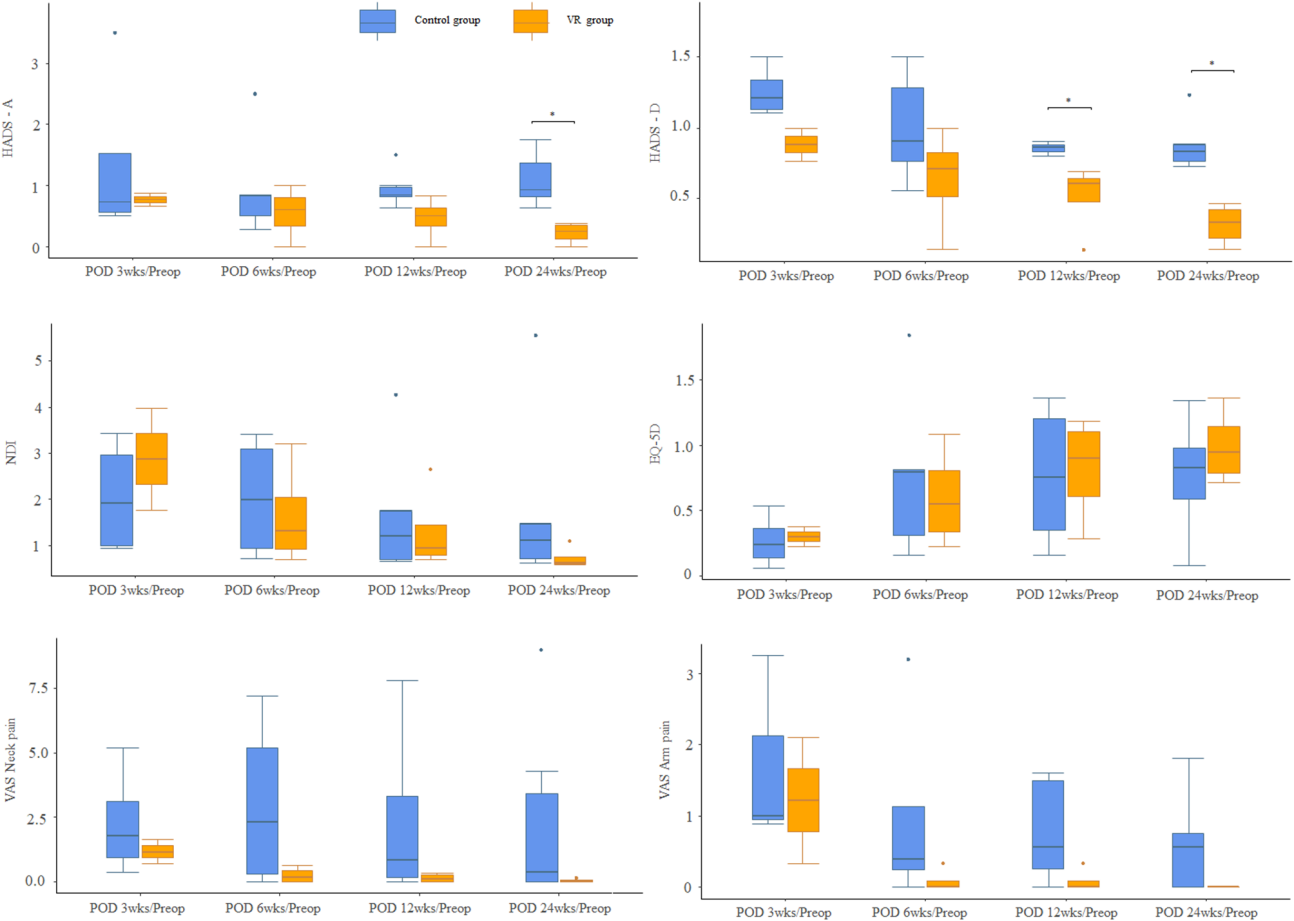
Proportions of patients with clinically meaningful changes in patient-reported outcomes at postoperative time points compared to the preoperative baseline. *HADS-A* Hospital Anxiety and Depression Scale - Anxiety, *HADS-D* Hospital Anxiety and Depression Scale - Depression, *NDI* Neck Disability Index, *EQ-5D* EuroQol 5-Dimension health questionnaire, *VAS* Visual Analog Scale, *Preop* Preoperative, *POD* postoperative day

The VR group demonstrated notable improvements in patient-reported outcomes compared with the Control group (Table 4; Fig. 2). (a) NDI: Functional disability scores improved significantly in the VR group compared with the Control group from preoperative values to 24 weeks postoperatively (40.0 [18.0–78.0] vs. 20.0 [12.0–22.0], *p* = 0.069). (b) EQ-5D index: Quality of life scores favored the VR group over the Control group at 24 weeks (0.5 [0.0–0.7] vs. 0.7 [0.6–0.8], *p* = 0.032). (c) VAS (pain): Significant reductions in arm pain were observed in the VR group compared with the Control group at 12 weeks (35.0 [0.0–80.0] vs. 0.0 [0.0–10.0], *p* = 0.048), with sustained results at 24 weeks. Neck pain reduction, though more pronounced in the VR group than in the Control group, was not statistically significant. (d) HADS: Anxiety (HADS-A) and depression (HADS-D) scores were lower in the VR group than in the Control group at 24 weeks, although the differences were not statistically significant. These findings suggest that VR-assisted rehabilitation provides both physical and psychological benefits for patients with C5 palsy (Table 4). Ratio analyses revealed significant improvements in HADS-D (depression subscale) at 12 and 24 weeks (12 weeks: VR: 0.6 [0.5–0.9] vs. Control: 0.9 [0.8–1.1], *p* < 0.05; 24 weeks: VR: 0.3 [0.2–0.6] vs. Control: 0.8 [0.6–1.0], *p* < 0.01). Similarly, the HADS-A (anxiety subscale) ratio at 24 weeks (POD 24 weeks/Preop) was significantly lower in the VR group than in the Control group (0.5 [0.3–0.7] vs. 0.9 [0.7–1.1], *p* < 0.05), indicating reduced anxiety.

**Table 4:** Comparison of patient–reported outcomes and muscle strength between control and VR groups

PROs & MRC scale	Preop				POD 3 wks				POD 6 wks				POD 12 wks				POD 24 wks				
	Control (*N* = 6)	VR (*N* = 4)	*P*	Cohen’s d	Control (*N* = 6)	VR (*N* = 4)	*P*	Cohen’s d	Control (*N* = 6)	VR (*N* = 4)	*P*	Cohen’s d	Control (*N* = 6)	VR (*N* = 4)	*P*	Cohen’s d	Control (*N* = 6)	VR (*N* = 4)	*P*	Cohen’s d	
NDI	31.0 (12.0–76.0)	25.0 (18.0–36.0)	> 0.999	0.59	52.0 (34.0–66.0)	68.0 (64.0–72.0)	0.247	1.461	48.0 (24.0–56.0)	44.0 (14.0–60.0)	0.902	0.256	41.0 (20.0–60.0)	31.0 (14.0–48.0)	0.334	0.656	40.0 (18.0–78.0)	20.0 (12.0–22.0)	0.069	1.481	
Equation 5D	0.6 (0.1–0.8)	0.8 (0.6–1.0)	0.285	0.967	0.1 (0.0–0.4)	0.2 (0.2–0.2)	0.481	0.143	0.2 (0.1–0.6)	0.5 (0.2–0.8)	0.389	0.446	0.4 (0.1–0.6)	0.7 (0.2–0.8)	0.069	0.957	0.5 (0.0–0.7)	0.7 (0.6–0.8)	0.032*	1.737	
VAS_Neck	15.0 (10.0–70.0)	70.0 (40.0–90.0)	0.067	1.635	50.0 (17.0–80.0)	53.0 (41.0–65.0)	> 0.999	0.172	46.0 (0.0–72.0)	10.0 (0.0–25.0)	0.262	1.141	29.5 (0.0–78.0)	4.0 (0.0–19.0)	0.322	1.074	21.5 (0.0–90.0)	0.0 (0.0–9.0)	0.337	1.07	
VAS_Arm	56.0 (0.0–90.0)	55.0 (30.0–70.0)	> 0.999	0.079	63.5 (51.0–80.0)	41.5 (20.0–63.0)	0.487	0.996	22.0 (0.0–64.0)	0.0 (0.0–10.0)	0.073	1.43	35.0 (0.0–80.0)	0.0 (0.0–10.0)	0.048*	1.739	29.5 (0.0–90.0)	0.0 (0.0–0.0)	0.072	1.336	
HADS-A/21	10.0 (2.0–21.0)	11.0 (4.0–18.0)	> 0.999	< 0.001	5.5 (4.0–14.0)	13.0 (12.0–14.0)	0.34	1.651	5.0 (2.0–18.0)	7.0 (0.0–12.0)	> 0.999	0.226	8.5 (3.0–18.0)	6.5 (0.0–9.0)	0.392	0.791	10.0 (3.0–21.0)	3.5 (0.0–6.0)	0.109	1.399	
HADS-D/21	12.0 (7.0–21.0)	10.5 (7.0–17.0)	0.667	0.305	11.0 (9.0–17.0)	13.0 (13.0–13.0)	0.481	0.397	12.0 (5.0–19.0)	9.0 (1.0–11.0)	0.27	0.845	10.0 (6.0–19.0)	7.0 (1.0–10.0)	0.241	1.04	9.5 (6.0–21.0)	4.0 (1.0–7.0)	0.067	1.541	
MRC grade for flexion	5.0 (5.0–5.0)	5.0 (5.0–5.0)	> 0.999		3.0 (2.0–4.0)	2.5 (2.0–3.0)	0.556	0.497	3.0 (2.0–5.0)	3.0 (2.0–5.0)	0.909	0.072	3.5 (3.0–5.0)	4.0 (3.0–5.0)	0.568	0.408	5.0 (4.0–5.0)	5.0 (4.0–5.0)	0.878	0.183	
MRC grade for abduction	5.0 (5.0–5.0)	5.0 (5.0–5.0)	> 0.999		3.0 (2.0–4.0)	3.0 (2.0–4.0)	0.817	0.212	3.0 (3.0–4.0)	3.0 (3.0–4.0)	0.878	0.183	4.0 (3.0–5.0)	4.5 (4.0–5.0)	0.556	0.497	5.0 (4.0–5.0)	4.5 (4.0–5.0)	0.350	0.667	

Values are mean rank (min-max)

*NDI* neck disability index, *Eq. 5D* EuroQol-5 dimension scale, *VAS* visual analog scale, *HADS*-A hospital anxiety and depression scale - anxiety subscale, *HADS*-D hospital anxiety and depression scale - depression subscale, *MRC* Medical Research Council, *POD* postoperative day, *Preop* preoperative day 1, *wks* weeks, *FI* fatigue index, *VR* virtual reality, *min* minimum, *max* maximum, *PROs* patient-reported outcomes. *, *P* < 0.05

For other patient-reported outcomes, including the NDI, EQ-5D, and VAS scores for neck and arm pain, ratio analyses did not reveal statistically significant differences between the groups at any postoperative time point. However, trends generally favored the VR group over the Control group. For instance, the NDI ratio at 24 weeks (POD 24 weeks/Preop) was lower in the VR group than in the Control group (0.5 [0.4–0.6] vs. 0.7 [0.6–0.8]), and the EQ-5D ratio was higher in the VR group than in the Control group (1.4 [1.3–1.5] vs. 1.2 [1.1–1.3]) (Fig. 2).

### VR-specific rehabilitation metrics

In the VR group, rehabilitation performance metrics showed significant improvements over time, reflecting enhanced motor coordination and engagement throughout the program. Patients participated in gamified VR exercises, where they placed virtual apples into baskets at varying heights. Performance metrics were recorded at each follow-up interval.

**Fig. 3:**
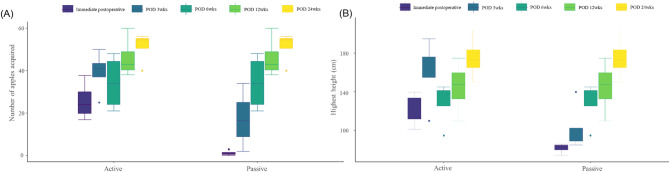
Changes in performance metrics over time in the VR rehabilitation group, comparing active and assisted modes. (A) Number of apples collected and (B) maximum height of the paralyzed arm elevation. *POD* Postoperative day, *wks* weeks

At the start of the program, the maximum height reached by the affected hand was 90.0 cm (median, range 80.0–95.0 cm). By 24 weeks postoperatively, this height increased to 145.0 cm (median, range 130.0–160.0 cm). Similarly, the number of successfully placed apples increased substantially over time. Initially, the median number of apples placed in the basket during a single cycle was 25 (range: 20–30). By 24 weeks, this number increased to 55 (range, 50–60), with most patients achieving near-maximal success rates (Fig. 3).

## Discussion

C5 palsy following cervical spine decompression or fusion surgery presents significant clinical and psychological challenges. Despite preoperatively counseling about the potential risks of such complications, the development of C5 palsy often induces substantial anxiety and distress [4, 5, 9]. This psychological burden arises from the sudden and often unexpected weakness in the deltoid and biceps muscles, which severely limits shoulder abduction and flexion, impairing daily activities and overall quality of life [3]. However, the pathophysiology of C5 palsy is not fully understood, with proposed mechanisms including direct nerve root traction, ischemic injury, and reperfusion injury [1, 9]. These factors complicate the development of targeted rehabilitation strategies.

Traditional rehabilitation approaches have often been passive, focusing on basic strengthening and range of motion exercises without sufficient stimulation to promote neural plasticity and muscle re-education [37]. In contrast, this study introduces VR-assisted rehabilitation as a novel approach to enhance motor recovery and alleviate the psychological burden associated with C5 palsy. One important consideration is that the VR group received approximately one additional hour of rehabilitation per session compared to the control group. Prior research has demonstrated that greater physical therapy duration is associated with improved functional outcomes, as noted in a systematic review by Peiris et al. [38]. Therefore, it is possible that the enhanced outcomes observed in the VR group may be partly due to the increased duration of task-specific motor activity, rather than the VR modality per se. However, unlike the therapist-supervised sessions analyzed in that review, both groups in our study received only education-based home rehabilitation, and only the VR group was provided with interactive, gamified feedback and progressive task difficulty. This distinction suggests that both duration and qualitative differences in rehabilitation approach may have contributed to the improved outcomes. Research has indicated that VR technology has the potential to revolutionize rehabilitation by providing an immersive and interactive environment that engages multiple sensory modalities, including visual, auditory, and proprioceptive feedback. Multisensory engagement enhances neuroplasticity and facilitates effective motor learning [13, 14, 19].

In this study, patients in the VR group exercised with real-time feedback, enabling them to track their progress and adjust their efforts accordingly. These patients demonstrated significant improvements in muscle activity compared with the Control group, likely due to enhanced neuromuscular performance. Although the VR program did not directly improve the MVIC of the deltoid muscle in patients with C5 palsy, the observed reduction in %MVIC in the VR group, when accompanied by improved MRC grades and shoulder function, may suggest more coordinated or efficient neuromuscular activation [39]; however, this interpretation should be viewed cautiously and warrants further study. These findings underscore the ability of VR to deliver targeted, high-intensity neurostimulation, which is crucial for recovering functional muscle activity and may contribute to improved performance in daily activities. These results align with previous studies demonstrating the efficacy of VR in enhancing motor function in various neurological conditions [40].

However, the lack of improvement in MVIC itself may be attributed to considerable interindividual variability, sparse assessment intervals, and the small sample size of this pilot study, which limits the generalizability of the findings. Future research with larger sample sizes is needed to explore whether VR programs significantly impact MVIC and FI and to identify the optimal timing for VR implementation following injury.

Although this study did not quantify patient engagement or motivation, the VR group’s favorable trend in HADS scores may reflect a greater emotional receptivity to the rehabilitation process, as previously reported in studies exploring gamified therapies [19, 39, 41]. Interactive tasks, such as “putting apples in the basket,” not only provided physical challenges but also made the exercises more enjoyable and engaging, leading to better adherence and improved outcomes. Beyond the physical benefits, the psychological advantages of VR-assisted rehabilitation are noteworthy. Patients with C5 palsy often face uncertainty regarding the extent of their paralysis and prospects for recovery. This uncertainty, coupled with the variable timeline for neurological recovery–which can range from weeks to months–contributes to heightened anxiety and a risk of depression [42]. Thus, addressing these psychological aspects is crucial. The HADS is particularly relevant in this context, with HADS-A assessing anxiety and HADS-D evaluating depression [43]. In this study, the VR-assisted rehabilitation group showed a notable reduction in both HADS-A and HADS-D scores compared with the control group, indicating its effectiveness in alleviating psychological distress during recovery. We posit that the immersive nature of VR likely distracted patients from their discomfort and disability, fostering a more positive rehabilitation experience. Furthermore, the ability of VR to provide a safe and controlled environment for practicing motor skills can boost patient confidence and reduce psychological barriers to recovery [44, 45].

### Limitations

This study has several limitations. First, the small sample size limits both the generalizability and statistical power of the results. Due to the low incidence of postoperative C5 palsy and the challenges of enrolling affected patients, who are often physically and emotionally vulnerable, this study was conducted as a preliminary pilot trial without a predefined power calculation. While appropriate statistical methods, including non-parametric tests, were applied, the findings should be interpreted with caution and viewed as an initial step toward future, larger-scale research. Second, group-level baseline imbalances may have influenced the outcomes. The VR group included relatively younger patients and had fewer comorbidities, notably a complete absence of diabetes, which could independently contribute to improved recovery. Although no statistically significant differences were observed in baseline characteristics (Table 1), such clinical imbalances are relevant and may confound outcome interpretation. Given the small sample size and exploratory nature of this pilot study, random variability in group composition was unavoidable. These limitations highlight the need for cautious interpretation and underscore the importance of future trials with larger, randomized cohorts and stratified or covariate-adjusted analyses. Third, although both groups received standardized rehabilitation education comprising range-of-motion exercises, the VR group received an additional hour of guided intervention. This discrepancy in total therapy dosage may have contributed to outcome differences beyond the VR modality itself. Fourth, while preoperative electrodiagnostic testing helped exclude patients with underlying neuromuscular diseases, the interpretation of surface EMG findings, such as %MVIC and MDF, as indicators of neuromuscular efficiency and fatigue remains exploratory. These parameters should be validated in larger, more homogeneous cohorts with appropriate control comparisons. Fifth, the use of a single VR system limited the frequency and customization of interventions. Future studies may benefit from more flexible VR platforms that allow frequent, home-based rehabilitation supported by web or Bluetooth interfaces, enabling remote monitoring and tailored feedback. Lastly, the rapid advancement of VR and digital rehabilitation technologies presents both opportunities and challenges. Integration with artificial intelligence and machine learning could support adaptive, personalized rehabilitation strategies that enhance patient engagement and functional recovery.

## Conclusions

This pilot study suggests that VR-assisted rehabilitation may offer potential benefits for improving motor function and emotional well-being in patients with postoperative C5 palsy, though larger, adequately powered studies are necessary to confirm these findings.

## Supplementary Information

Below is the link to the electronic supplementary material.

Supplementary Material 1.

Supplementary Material 2.

## Data Availability

The data that support the findings of this study are available from the corresponding author, upon reasonable request.
