# Supplementary material for: Virtual reality-assisted rehabilitation for postoperative C5 palsy: a pilot exploratory randomized controlled trial
Source: J Neuroeng Rehabil. 2025 Aug 13;22:178. doi: 10.1186/s12984-025-01716-7 (PMC12345129; doi:10.1186/s12984-025-01716-7)
Supplement: Supplementary file 1 — Supplementary Material 1. [file 12984_2025_1716_MOESM1_ESM.docx]

**Supplementary Video Footnote**

Supplementary Video 1. Demonstration of active and assisted VR rehabilitation modes for postoperative C5 palsy.
